# Influence of Rhenium Content on Vacancy-Type Defect Distribution in Mo–Re Alloys Under Room-Temperature Irradiation

**DOI:** 10.3390/ma19122632

**Published:** 2026-06-18

**Authors:** Yongli Liu, Qigui Yang, Yunpeng Zhou, Tong Fu, Linjiang Chai, Xingzhong Cao

**Affiliations:** 1 The First Sub-Institute, Nuclear Power Institute of China (NPIC), Chengdu 610005, China; 2 Institute of High Energy Physics, Chinese Academy of Sciences (CAS), Beijing 100049, China; 3 College of Materials Science and Engineering, Chongqing University of Technology (CQUT), Chongqing 400054, China

**Keywords:** molybdenum–rhenium alloy, positron annihilation spectroscopy, vacancy-type defects, depth distribution, rhenium effect

## Abstract

Mo–Re alloys serve as critical structural components for high-temperature nuclear reactors, and their irradiation degradation is closely related to the evolution of vacancy-type defects. In this study, heavy-ion and He-ion irradiations were performed under RT to introduce an average displacement damage of 3.5 dpa within the 1 μm-thick surface layer of Mo–Re alloys with Re content up to 47 wt.%. PALS, SPB-DBS and CDB techniques were employed to characterize the size, concentration, depth distribution and local chemical environment of irradiation-induced vacancy-type defects. The results demonstrate that the longer lifetime component of irradiated Mo–Re alloys ranged from 262 to 280 ps, corresponding to medium-sized vacancy clusters. The *S* parameter of all specimens increased significantly from approximately 0.42 to 0.50, with negligible differences (<0.01) among various Mo–Re alloys. No distinct characteristic peak of Re was observed near 17 × 10^−3^ m_0_c at the vacancy sites, which was inconsistent with simulation predictions. Mo–Re alloys exhibit similar vacancy-type defect features to pure Mo, implying weak interactions between Re solute atoms and vacancy-type defects under RT irradiation.

## 1. Introduction

In the field of deep-space exploration, energy and power supply remain one of the critical challenges. Extreme environmental conditions impose rigorous requirements on space power systems, such as safety, reliability, power-to-mass ratios, stable and sustained power generation, and efficient energy storage. In the 1960s, deep-space spacecraft were mostly designed to be compact and highly functional, with high-temperature reactors widely adopted as their power systems [1,2,3,4,5,6]. These high-temperature structural components deployed in such systems were required to possess excellent long-term thermal creep resistance, favorable fabricability, a low ductile-to-brittle transition temperature (DBTT), good weldability, low density, strong resistance to embrittlement induced by oxygen, carbon dioxide, and nitrogen, and suppressed irradiation swelling, as well as well-characterized mechanical and thermophysical properties [7]. In space power systems, heat-resistant structural components operate at 1000–1500 K under near-atmospheric or slightly elevated pressures in liquid alkali-metal-cooled reactors (e.g., heat-pipe systems), and at 1100–1400 K under pressures well above 2 MPa in gas-cooled reactors [7,8]. Under such extreme high-temperature conditions, refractory tungsten–rhenium (W–Re) alloys and molybdenum–rhenium (Mo–Re) alloys are adopted as candidate structural materials owing to their superior properties including high melting points, good thermal conductivity, strong corrosion resistance, and a low sputtering rate [9,10,11,12]. Compared with W–Re alloys, Mo–Re alloys are more favorable for the design of these components, which are attributed to their lower induced activation rate, lower density, good creep resistance, and ductility [13], as well as excellent temperature compatibility with ceramic fuels (e.g., UO_2_: 1550 K, UN: 1300 K, UC: 1250 K [14]) and alkali metal coolants (e.g., Na: 1400 K, Li: 1700 K [15]). In addition, as strong resonance absorbers of thermal and epithermal neutrons, Re atoms act as an excellent neutron-spectrum-shifting material [4], ensuring that the space power system maintains a sufficiently deep subcritical state under accident conditions. Therefore, Mo–Re alloys have attracted extensive attention worldwide, and their service performance is crucial for the operational lifetime of these power systems.

Alloying with Re effectively increases the low-temperature ductility of Mo, while significantly enhancing the creep strength (1.0% creep deformation for pure Mo, 3 × 10^−4^ for Mo–14 wt.% Re and nearly 0% for Mo–46 wt.% Re after 7 years at 1400 K/7 MPa [15]) and the oxygen embrittlement resistance. Additionally, Re addition elevates the recrystallization temperature (~1200 K for pure Mo, ~1645 K for Mo–14 wt.% Re, ~1575 K for Mo–46 wt.% Re [15]) and reduces the DBTT (~340 K for pure Mo, ~173 K for Mo–5 wt.% Re, ~140 K for Mo–14 wt.% Re, ~75 K for Mo–46 wt.% Re [7,15]). However, in high-flux neutron environments, irradiation-induced embrittlement remains one of the primary issues for Mo–Re alloys, which arises from the evolution of irradiation-induced microstructural damage and the formation of Re-rich precipitates. Yet the intricate interactions between Re atoms and irradiation-induced defects, together with their underlying physical origins, have not been fully clarified so far.

The microstructural evolution of neutron-irradiated Mo–Re alloys exhibits a strong dependence on Re concentration. Hasegawa et al. [16] confirmed that Mo–5Re and Mo–41Re alloys display distinctly different damage behaviors under neutron irradiation: voids readily nucleate in Mo–5Re at 646–1073 K, dislocation loops from above 646 K, and fine needle-like Re-rich precipitates at temperatures exceeding 792 K. In contrast, the precipitation onset temperature for Mo–41 wt.% Re is 679 K. With increasing temperature, the number and size of precipitates rise markedly, whereas void dimensions and number density remain generally lower relative to Mo–5Re. Chakin et al. [17] reported that rising Re content reduces the number density of 7.5–10 nm average-sized dislocation loops by a factor of 4–6, with complete suppression of dislocation-loop formation within the weld fusion zone of Mo–41Re. Nemoto et al. [18] performed neutron irradiation on pure Mo and Mo–Re alloys containing 2–41 wt.% Re at 681–1072 K, and observed void formation in all specimens. The average void size increases with rising Re content and irradiation temperature, accompanied by a decrease in void number density. Notably, the void swelling of Mo–41Re is only approximately 0.1%, remarkably lower than that in low-Re alloys. Krajnikov et al. [19] found that under low-temperature neutron irradiation (373–433 K), both the size and number density of dislocation loops decline with increasing Re concentration. In situ TEM characterizations have revealed the evolution of dislocation loops in pure Mo and Mo–5Re alloys during ion irradiation: dislocation loops nucleate in Mo–5Re at the early irradiation stage, with their number density increasing continuously before undergoing coalescence, leading to larger loop sizes and lower number densities [8,20,21]. Cui et al. [22,23] systematically clarified the origin and evolution mechanism of dislocation loop punching in irradiated Mo–5Re alloys, and demonstrated that the nucleation and growth of dislocation loops are driven entirely by bubble evolution at temperatures above ~0.4T_m_. Most studies indicate that the dominant irradiation defects in Mo–Re alloys are voids and dislocation loops at low temperatures, whereas brittle Re-rich σ and χ phases tend to precipitate at high temperatures. The size of these precipitates increases with rising Re content and irradiation temperature, accompanied by a gradual reduction in their number density. Under irradiation at 1072 K, the volume fraction of σ phase in Mo–41Re can reach up to 26% [18]. On this basis, Lu et al. [8] employed first-principles calculations and static molecular dynamics simulations to confirm that the V_2_–Re_1_ complex acts not only as a key nucleus for vacancy cluster formation but also as a critical precursor for subsequent vacancy aggregation, Re segregation and phase precipitation. It is evident that the interactions among vacancies, self-interstitial atoms (SIAs), and solute Re atoms dominate the irradiation-induced microstructural evolution and radiation embrittlement of Mo–Re alloys over the entire temperature range. Therefore, systematic investigations of the interactions between vacancies and Re atoms are essential to elucidate the temperature-dependent mechanisms underlying irradiation defect evolution at varying Re concentrations and to clarify the formation pathways of Re-rich clusters and precipitates.

To address this issue, a series of Mo–Re alloys with Re content up to 50 wt.% were selected for this study. Sequential heavy-ion and helium (He) irradiation were conducted at room-temperature (RT) to introduce a uniform damage distribution within near-surface region (~1 μm) of specimens. By combining positron annihilation spectroscopy (PAS) techniques, including positron annihilation lifetime spectroscopy (PALS), Doppler broadening spectroscopy (DBS), and coincidence Doppler broadening spectroscopy (CDB), together with transmission electron microscopy (TEM), the influence of Re content on the distribution of vacancy-type defects in Mo–Re alloys at the early irradiation stage was systematically investigated.

## 2. Experimental Details

### 2.1. SRIM Simulation

**Figure 1:**
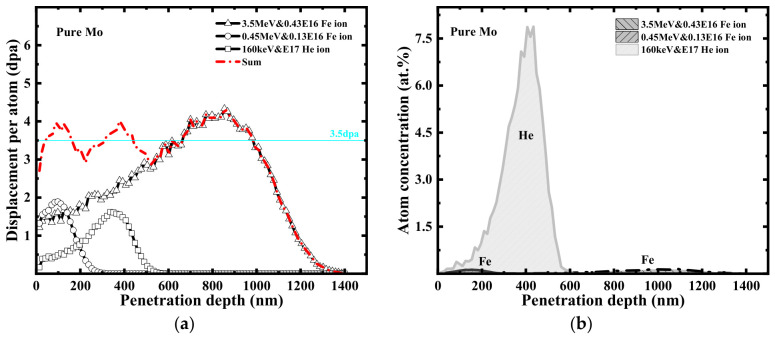
SRIM simulation results of pure Mo subjected to sequential ion irradiations (Fe+Fe+He). (**a**) Depth profile of displacement damage, (**b**) Depth profile of He atom concentration.

Figure 1a,b present the depth distributions of displacement damage and He concentration in pure Mo subjected to sequential ion irradiation, as simulated by SRIM-2008 (Stopping and Range of Ions in Matter) [24]. In this work, the Kinchin–Pease formalism-based quick calculation option [25,26] was chosen for direct comparison between ion (Z ≥ 2) and neutron data. Notably, for ions with Z ≤ 2 (e.g., protons), the full damage cascade model should be adopted. The displacement threshold energy of Mo and Re was set to 60 eV [27], and the lattice binding energy was set to 0 eV [25,28]. To achieve damage characteristics comparable to neutron irradiation conditions, sequential multi-beam ion irradiation was employed to produce a nearly uniform radiation damage distribution. The irradiation sequence comprised the following: first, 3.5 MeV Fe ions to a fluence of 4.3 × 10^15^ ions·cm^−2^; then, 0.45 MeV Fe ions to a fluence of 1.3 × 10^15^ ions·cm^−2^; and finally, 160 keV He ions to a fluence of 1.0 × 10^17^ ions·cm^−2^. All ion beams were implanted perpendicular to the polished single-side specimen surface. SRIM simulations show that irradiation-induced displacement damage remains nearly constant across a depth of 1000 nm, with average damage, with average damage level fluctuating around 3.5 dpa. He atoms mainly deposit within 200–600 nm, reaching a peak atomic concentration of approximately 7.87 at% at a depth of 434 nm. The corresponding calculation is defined by the formula given below [29]:

(1) $$dpa=\frac{N_{d}\Phi }{D_{s}}$$

(2) $$at.\%=\frac{N_{i}\Phi }{D_{s}}\times 100$$

(3) $$D_{s}=\frac{\rho N_{A}}{A}$$

where $N_{d}$ is the number of vacancies generated by incident and recoil ions in the material, $N_{i}$ is the number of He atoms deposited per unit distance. Both parameters can be extracted from SRIM output files. Φ is the ion fluence. $D_{s}$ is the atomic density of Mo which can be derived from the atomic density ρ, atomic mass A and Avogadro’s constant $N_{A}$. The predicted damage level and He concentration are relatively higher than the experimental results because the simulation ignores recombination [30,31,32].

### 2.2. Sample Preparation

The chemical compositions of all specimens (10 mm × 10 mm × 1 mm) are presented in Table 1. The impurity elements in the specimens include C, N, O, P, Bi, Sn, Al, Fe, Cu, Ni, Si, etc., with the total impurity content lower than 0.01 wt.%. The deviation of the main alloying elements does not exceed ±3 wt.%. All specimens were mechanically polished using 180, 360, 600, 800, 1500, and 2000-grit sandpapers to obtain a flat surface. Subsequently, the specimens were further polished to a mirror finish using 9 μm, 3 μm, and 1 μm SiO_2_ polishing suspensions. Finally, vibratory polishing was performed for residual stress relief, yielding a surface roughness below the micrometer scale. For vibratory polishing, 0.05 μm Al_2_O_3_ polishing fluid was adopted, with a vibration frequency of 40 Hz and a polishing duration of 4 h. Afterwards, all specimens were subjected to recrystallization annealing at 1773 K for 1 h under a vacuum of 1.2 × 10^−5^ Pa [13,19].

**Figure 2:**
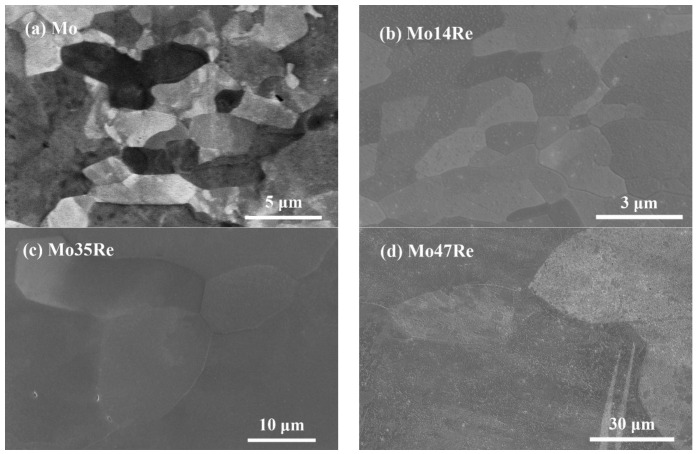
Surface morphologies of annealed (**a**) pure Mo, (**b**) Mo14Re, (**c**) Mo35Re, and (**d**) Mo47Re alloys prior to irradiation.

Figure 2 shows the surface morphologies of the annealed specimens. The average grain size of pure Mo after annealing is approximately 6 μm, while that of Mo–14Re is about 5 μm. As the Re content increases to 35 wt.% and 47 wt.%, the corresponding average grain sizes reach approximately 37 μm and 64 μm, respectively. In Mo–Re alloys, Re acts as a potent solid-solution strengthener. The elevated temperature during recrystallization annealing provides sufficient thermal driving force to promote extensive grain nucleation and coarsening via continuous grain boundary migration. With increasing Re content, the driving force for grain coalescence is further enhanced, leading to pronounced grain coarsening and the formation of ultra-large grains in Mo47Re. A higher Re content elevates the recrystallization temperature of the alloy, thereby facilitating abnormal grain growth during high-temperature annealing.

### 2.3. Ion Irradiation

Ion irradiation experiments on Mo, Mo14Re, Mo35Re and Mo47Re alloys were conducted at the 320 kV Platform for Multidisciplinary Research at the Institute of Modern Physics, Chinese Academy of Sciences (CAS). The ion beam was incident perpendicularly to the sample surface, with specimens affixed to the irradiation target holder via conductive carbon tape applied to their backsides. Irradiation was performed at RT under a target chamber vacuum of 5 × 10^−4^ Pa. As detailed in Table 2, all annealed specimens underwent sequential ion irradiations: first, Fe-ion irradiation at 3.5 MeV (Step I) with a total fluence of 4.3 × 10^15^ ions·cm^−2^; followed by Fe-ion irradiation at 0.45 MeV (Step II) with a total fluence of 1.3 × 10^15^ ions·cm^−2^; and finally, He^2+^ irradiation at 0.16 MeV (Step III) with a total fluence of 1 × 10^17^ ions·cm^−2^.

## 3. Characterization Techniques

### 3.1. Positron Annihilation Lifetime Spectroscopy (PALS)

The positrons commonly used in laboratories are derived from the positive β decay of ^22^Na, which emits fast positrons with a maximum energy of 0.545 MeV at a relative intensity of 90.2% and almost simultaneously emits 1.27 MeV γ photons. After the fast positrons enter a substance, they slow down to the thermal energy region within a few picoseconds. During diffusion, they annihilate with electrons to produce two 0.511 MeV γ photons. The average time elapsed from the emission of a positron to its annihilation in the specimen is defined as the positron annihilation lifetime. Open-volume defects such as vacancies in crystals can trap positrons (due to the low electron density at defect sites), resulting in delayed annihilation and increased annihilation lifetime. The magnitude of the lifetime can reflect the size characteristics of vacancy-type defects in the material.

### 3.2. Doppler Broadening Spectroscopy (DBS)

When electron–positron pair annihilation occurs, the positron is assumed to have zero momentum, while electrons in the material still possess momenta of several eV. This causes the two γ photons produced by annihilation to undergo an energy shift relative to 511 keV. The Doppler broadening spectrum of a perfect crystal lattice exhibits an inverted parabolic profile. In contrast, for defective specimens, positron annihilation with core electrons follows a Gaussian distribution, whereas annihilation with valence electrons yields an inverted parabolic shape. In experiments, line-shape parameters such as the *S* parameter (ratio of photons from positron annihilation with low-momentum valence electrons to the total photon count) and the *W* parameter (ratio of photons from positron annihilation with high-momentum core electrons to the total photon count) are commonly used to characterize the positron annihilation behavior. When positrons are trapped by defects, the probability of annihilation with high-momentum core electrons decreases, and the *S* parameter increases accordingly. Defect information in the material can be obtained by comparing the variations in the *S* and *W* parameters measured under identical experimental conditions.

### 3.3. Coincidence Doppler Spectroscopy (CDB)

During DBS measurements, one of the 511 keV γ photons associated with the transverse momentum component of the annihilating electron–positron pair is detected using a high-purity germanium (HPGe) detector. The detector exhibits an energy resolution of approximately 1.5 keV (full width at half maximum, FWHM) at 511 keV, corresponding to a momentum resolution of 5.87 × 10^−3^ m_0_c. This method yields a line-shape curve of photon energy versus count, with a peak-to-background ratio of 150:1. In this case, the photon counts in high-energy windows—regions sensitive to elemental information—are swamped by intense background signals. To overcome this limitation, the two-dimensional Doppler broadening spectrum acquired with a two-detector coincidence system suppresses the background count rate by three orders of magnitude and improves the peak-to-background ratio. Consequently, analysis of the coincident Doppler broadening spectrum enables the characterization of the chemical environment surrounding lattice defects.

### 3.4. Slow Positron Beam (SPB)

The average energy of positrons emitted by a ^22^Na source exceeds 260 keV. The positron penetration range can be calculated using the formula $depth=(40E^{1.6})/\rho$, where ρ is the material density (g·cm^−3^) and E is the positron energy (keV). Calculations indicate that the maximum penetration range of positrons in pure Mo reaches approximately 93 μm, with an average range of around 28 μm. The intrinsic energy spectrum of the positron source covers a broad energy span. To characterize near-surface defects in materials, a moderator is employed to slow down positrons, producing a monoenergetic and energy-adjustable positron beam with energies reduced to several eV to tens of eV.

### 3.5. The Transmission Electron Microscope Based on Focus Ion Beam (FIB-TEM)

For transmission electron microscopy (TEM) characterization, extremely thin specimens (<100 μm) need to be prepared. A focused ion beam (FIB) was adopted to fabricate local cross-sections of the specimen. Liquid gallium (Ga) serves as the ion source due to its low melting point, low vapor pressure, and excellent oxidation resistance. Liquid gallium is confined to a fine tip by an electric field, and the extracted Ga ion beam is used to cut the sample surface, finally obtaining a thin area of about 4 × 3 μm^2^ for TEM observation [33].

## 4. Results and Discussion

### 4.1. Defect Type in RT-Irradiated Mo–Re Alloys

All measured PAL spectra can be decomposed into three lifetime components through analysis with the LT-9.0 program: $\tau_{1}$ (the short-lifetime component), $\tau_{2}$ (the long-lifetime component), and $\tau_{3}$ (third-lifetime component). As shown in Table 3, $\tau_{3}$ represents the positron annihilation lifetime of the radiation source itself, sample surface, and boundaries. When positrons penetrate the Kapton film, some positrons annihilate within the film due to interfacial backscattering, diffusion return, and re-emission from the sample surface which has a negative work function. This makes $\tau_{3}$ larger than the other two components. Specifically, $\tau_{3}$ is approximately 1.9–2.3 μs in irradiated Mo–Re alloys. During the fitting of raw data, this source-surface component is deducted from the spectral data, leading to an extremely low intensity (<1%) of $\tau_{3}$. It fluctuates moderately between 0.6 and 1% and remains nearly constant across different specimens. For these reasons, $\tau_{3}$ can generally be neglected in PALS analysis throughout this work.

In the field of PALS, each positron annihilation lifetime parameter represents a weighted average of multiple electronic states. Positrons annihilate in the bulk free state, where the free-state annihilation rate $\lambda_{f}$ is determined by the material’s electronic structure characteristics. When defects such as vacancies replace lattice atoms, these defect sites are equivalently negatively charged. Positrons are more likely to be trapped by these defects, with the positron annihilation rate in the defect-trapped state denoted as $\lambda_{d}$. The positron annihilation lifetime in the matrix is denoted as $\tau_{1}$, and that of positrons trapped at vacancy-type defects is denoted as $\tau_{2}$. The average positron lifetime, which is denoted as $\tau_{m}$, is a weighted average of each lifetime component, which characterizes the overall defect state of the specimen. $I_{1}$ and $I_{2}$ represent the relative intensities corresponding to $\tau_{1}$ and $\tau_{2}$, respectively.

**Figure 3:**
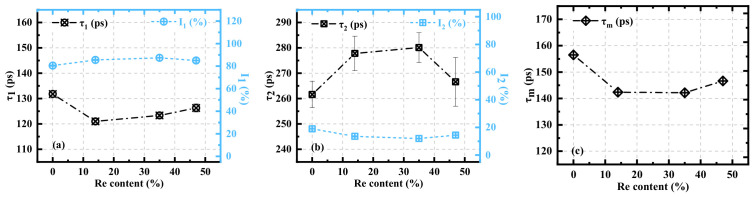
PALS results for irradiated pure Mo, Mo14Re, Mo35Re, and Mo47Re: (**a**) $\tau_{1}$ and $I_{1}$, (**b**) $\tau_{2}$ and $I_{2}$, (**c**) average positron lifetime $\tau_{m}$.

Figure 3 presents the PALS results of irradiated pure Mo, Mo14Re, Mo35Re, and Mo47Re alloys. The raw PAL spectra of all specimens are provided in Appendix A. The bulk lifetime of defect-free pure Mo ranges from 103 to 106 ps, which is close to that of tungsten at approximately 105 ps [34]. Through calculation, the positron annihilation lifetime of monovacancies, divacancies, trivacancies in pure Mo is about 205 ps, 223 ps, 239 ps. The lifetime at defect sites increases gradually with the aggregation and cluster size growth of vacancies. Specifically, the lifetime reaches 325 ps for 9-vacancy clusters, and stabilizes at approximately 420 ps for large-vacancy aggregates containing more than 30 vacancies. In contrast, both the bulk lifetime and monovacancy lifetime of pure Re are 14–17 ps lower than those of pure Mo. Theoretically, the dissolution of Re atoms into the Mo matrix modulates the local electronic structure and can reduce the bulk lifetime of Mo–Re alloys. As shown in Figure 3a, the $\tau_{1}$ of irradiated Mo is approximately 130 ps, distinctly higher than 106 ps for the defect-free Mo matrix. Continuous ion irradiation introduces lattice distortion, residual internal stress, and dispersed intrinsic point defects within the near-surface region. Such matrix damage alters the distribution of free electrons and prolong the annihilation time of free positrons, thereby leading to an overall elevation of $\tau_{1}$ in all irradiated specimens. When the Re content increases to 14 wt.%, $\tau_{1}$ decreases to 121 ps. As the Re content further rises to 35 wt.% and 47 wt.%, high-concentration solute atoms induce relatively severe lattice distortion, thereby leading to a slight increase in $\tau_{1}$. The matrix annihilation intensity $I_{1}$ remains in the range of 80–85% for all specimens, demonstrating that positron annihilation predominantly occurs in the matrix.

Figure 3b shows that the $\tau_{2}$ of all specimens (262–280 ps) are significantly higher than the characteristic lifetime of monovacancies in pure Mo (~205 ps), while remaining far below the lifetime of large V_30_ clusters (~420 ps). These measured lifetimes fall within the range of calculated positron annihilation lifetimes of V_2_–V_6_ vacancy clusters (about 223 ps, 239 ps, 265 ps, 281 ps, 303 ps). This indicates that the dominant irradiation-induced defects in Mo–Re alloys are neither isolated monovacancies nor large-scale vacancy aggregates, but rather medium vacancy clusters and vacancy-impurity complexes. Recent research by Hu et al. [34]. reported that as irradiation damage accumulates up to 2 dpa in pure Mo, the fraction of isolated monovacancies decreases to approximately 23%, while the proportion of V_4_–V_9_ clusters rises markedly to around 24%. Divacancies and trivacancies remain the dominant defects, accounting for about 53% of total vacancies. DFT calculations reveal that monovacancy migration is markedly suppressed at room temperature relative to high-temperature conditions. Meanwhile, OKMC simulation from Hou et al. [35] further demonstrate that long-range diffusion of vacancies remains restricted at 300 K. Nevertheless, the extreme vacancy concentration inside individual collision cascade cores facilitates short-range vacancy agglomeration and coalescence via cascade overlap, eventually generating large vacancy clusters exceeding V_6_.

During ion irradiation, implanted He atoms readily combine with vacancies to form He–vacancy complexes, which reduce the effective open volume of defects. With increasing Re content, $\tau_{2}$ varies within a narrow range of 266–280 ps and is slightly higher than that of pure Mo. Meanwhile, the corresponding defect annihilation intensity $I_{2}$ gradually decreases from 19% to 12%. This indicates that Re atoms slightly reduce the density of vacancy-type defects, while their inhibitory effect on vacancy cluster migration and aggregation remains negligible at RT.

(4) $$\lambda_{1}=1/\tau_{1}=\lambda_{f}+K$$

(5) $$\lambda_{2}=1/\tau_{2}=\lambda_{d}$$

(6) $$\lambda_{f}=1/\tau_{f}=\frac{I_{1}\lambda_{1}+I_{2}\lambda_{2}}{I_{1}+I_{2}}$$

(7) $$\tau_{m}=\frac{I_{1}\tau_{1}+I_{2}\tau_{2}}{I_{1}+I_{2}}$$

(8) $$K=\frac{\lambda_{f}(\tau_{m}-\tau_{f})}{\left(\tau_{d}-\tau_{m}\right)}$$

The PALS results shown in Figure 3 are summarized in Table 3. According to Equations (4)–(8), the annihilation rate of the short-lifetime component ($\lambda_{1}$) is derived from both the free-state annihilation rate ($\lambda_{f}$) and the defect trapping-state annihilation rate (denoted as K). The calculated K values for pure Mo, Mo14Re, Mo35Re, and Mo47Re are 0.72, 0.64, 0.54, and 0.60 (×10^9^ s^−1^), respectively. An increase of K value indicates an elevated open-volume defect level in the material.

For pure Mo, the defect concentration remains relatively high after irradiation. However, as the Re content gradually increases to 35 wt.%, the K value exhibits a slight declining trend, indicating relatively lower open volume within the material. When the Re content rises to 47 wt.%, the K value no longer decreases, and the inhibitory effect of Re on defect density and size reaches a dynamic balance. As shown in Figure 3c, the average positron lifetime $\tau_{m}$, which characterizes the overall average open volume of the material, also decreases first and then rises slowly with increasing Re content. This demonstrates that low-concentration solute Re atoms can suppress the number of irradiation-induced defects to a certain extent at RT. According to DFT calculations reported by Hou et al. [35], the migration barrier of vacancy clusters in molybdenum rises to 2 eV as the cluster size increases up to V_15_, and thereafter stabilizes within the range of 1–2 eV for larger clusters exceeding V_15_. The migration barrier of monovacancy in pure Mo is about 1.2 eV, which limits the long-range migration of single vacancies at 300 K. Nearly all vacancy clusters nucleate in situ within high-density cascade-damaged regions generated by nonequilibrium ionic collisions. Meanwhile, these small vacancy clusters retain adequate mobility for further coalescence.

In contrast, high Re content (>35 wt.%) suppresses vacancy aggregation and elevates the number density of Re–vacancy and He–Re–vacancy complexes. These competitive synergies lead to a slight rebound in positron annihilation parameters K and $\tau_{m}$ of Mo47Re alloy. However, the overall fluctuation of positron annihilation lifetime is restricted below 20 ps for all specimens, revealing that Re exerts only a weak inhibitory influence on vacancy clustering at RT.

### 4.2. Defect Distribution in Mo–Re Alloys

**Figure 4:**
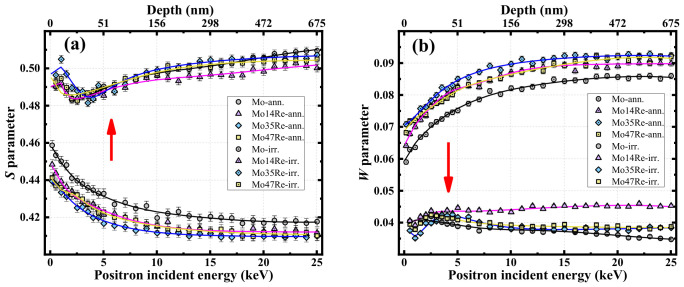
Depth dependence of (**a**) *S* parameter and (**b**) *W* parameter for the annealed and irradiated Mo, Mo14Re, Mo35Re, and Mo47Re specimens. The corresponding positron implantation depth is presented on the top horizontal axis. All solid lines are VEPFIT-fitted curves for the measured *S* and *W* parameter data. Red arrows represent RT ion irradiation.

Using the SPB-DBS technique with positron energies continuously adjustable from 0 to 25 keV, depth profiles of irradiation-induced vacancy-type defects in Mo, Mo14Re, Mo35Re, and Mo47Re were systematically investigated, as shown in Figure 4. All specimens exhibit the characteristic behavior of low *S* values in the annealed state and high *S* values after irradiation, as depicted in Figure 4a. The annealed specimens show generally low *S* parameters that gradually decrease with increasing positron incident energy before reaching a plateau. This confirms that high-temperature annealing effectively eliminates most intrinsic defects, resulting in a low and uniformly distributed defect concentration. After irradiation, the defect level increases significantly, with the *S* parameter rising from approximately 0.42 to 0.50. This indicates that irradiation introduces a high density of vacancy-type defects to a depth of at least 675 nm, which is consistent with the SRIM simulation results. Due to surface effects, all specimens exhibit a pronounced high-*S* surface damage layer at positron energies below 2 keV. As depth increases, the *S* parameter first decreases and then rises to a stable plateau, indicating that open-volume defects reach a saturated concentration beyond 600 nm. This saturation phenomenon originates from the dynamic competition among Frenkel pair recombination, cascade overlap, the agglomeration of self-interstitial loops, and the migration and coalescence of vacancy clusters. It reveals that a steady-state microstructure forms in the material once the irradiation damage accumulates to a specific threshold level [34].

**Figure 5:**
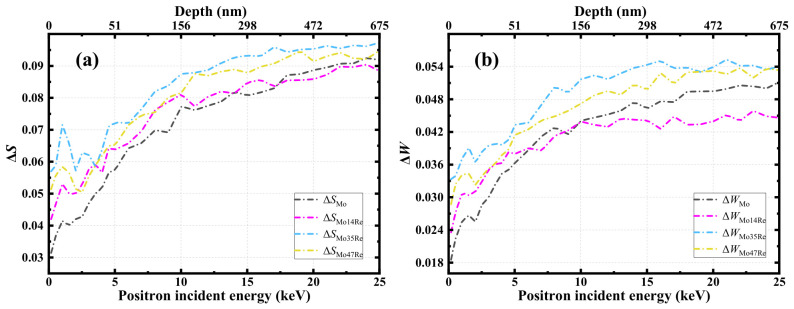
Depth dependence of Δ*S* (**a**) and Δ*W* (**b**) parameters for Mo, Mo14Re, Mo35Re, and Mo47Re alloys.

The effect of Re addition on the post-irradiation *S* parameter appears relatively minor. To clarify this effect, the Δ*S* parameter, defined as the difference between the *S* parameters of irradiated and annealed specimens, is introduced, regardless of vacancy-interstitial recombination or the formation of complex defects during irradiation. As shown in Figure 5a, Δ*S* values of Re-containing specimens are notably higher than those of pure Mo. This suggests that Re addition promotes the accumulation of vacancy-type defects at RT. Combined with the PALS results for $\tau_{2}$, this accumulation is mainly attributed to an increase in the size of vacancy cluster defects. Although the effect is modest, it again confirms that the inhibitory effect of Re is nearly negligible at RT. A similar phenomenon was also observed in our RT-H/He ion irradiation experiments on W3Re, W5Re, and W25Re alloys. Both indicate that Re exerts a negligible influence on the migration and coalescence of vacancy-type defects during RT irradiation. In contrast, Re significantly suppresses the accumulation of vacancy-type defects in W–Re alloys during high-temperature irradiation [36].

Figure 4b presents the depth-dependent *W* parameters of annealed and irradiated Mo–Re alloys, whose evolution shows a complementary relationship to that of the *S* parameter. The annealed specimens exhibit generally high *W* parameters ranging from 0.07 to 0.09 that increase slowly with depth before reaching a plateau, consistent with the low defect concentration in the matrix. After irradiation, the *W* parameters decrease significantly to the range of 0.035–0.045. This confirms that the introduction of numerous vacancy-type defects by irradiation reduces the proportion of high-momentum core electron annihilation and induces reconstruction of the local chemical environment. The magnitude of the post-irradiation decrease in *W* parameters increases gradually with Re content, indicating that the presence of Re can moderately reduce the high-momentum electron density around defects at RT. This effect is insignificant in low-Re systems and even exhibits an opposite trend at depths greater than 300 nm. As shown in Figure 5b, the variations in Δ*S* and Δ*W* for Mo14Re are comparable to those of pure Mo, and even smaller at greater depths. This indicates that Re additions below 14 wt.% only exert a weak inhibitory effect on the density of irradiation-induced defects at RT. In contrast, Re contents above 35 wt.% still show a promoting effect on the concentration of open-volume defects within the measured depth range under RT irradiation.

**Figure 6:**
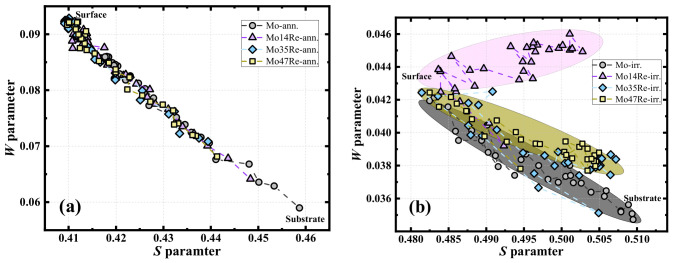
*S*–*W* trajectories of annealed (**a**) and irradiated (**b**) specimens for Mo, Mo14Re, Mo35Re, and Mo47Re alloys. The elliptical regions denote the approximate range of data distribution.

Figure 6 presents the *S*–*W* correlation trajectories for the annealed and irradiated Mo–Re alloys, which can be used to evaluate the evolution of dominant defect types [37,38]. The measured (*S*, *W*) data points falling approximately on a straight line generally indicate that no significant change in defect type occurs within the material. In contrast, deviations in the *S*–*W* trajectory indicate alterations in the defect type or local chemical environment [37,39]. As shown in Figure 6a, all data points of the annealed specimens are distributed approximately along a straight line, with a strong negative correlation between the *S* and *W* parameters. This indicates that the matrix is dominated by a single type of residual vacancy-type defects after high-temperature annealing, with no significant formation of complex defects. With increasing Re content, the data points exhibit a slight overall shift toward lower *S* and higher *W* values. This weak interaction between solute Re atoms and residual vacancies slightly affects the electron annihilation characteristics of the defects, yet does not alter the dominant defect type in the matrix.

As shown in Figure 4b, the *S*–*W* trajectories of the irradiated specimens exhibit distinct divergence. The overall distribution shifts toward higher *S* and lower *W* values, forming three clearly separated regions corresponding to different Re contents. For pure Mo, the data points lie along a straight line with a relatively steep slope, representing a “vacancy-type defect growth/increase” trend. This indicates that irradiation-induced defects in pure Mo are dominated by continuously growing vacancy clusters, with a relatively simple local chemical environment. For Mo14Re, the data points deviate significantly from the distribution line of pure Mo and form an independent region with higher *W* and lower *S* values. This confirms the formation of Re–vacancy complexes under irradiation at low Re content. Such defects reduce the effective open volume (consistent with the PALS results showing a decrease in defect number and an increase in defect size) while significantly increasing the proportion of high-momentum electron annihilation around the defects, thereby altering the local chemical environment.

The trajectories of Mo35Re and Mo47Re fall in the transition region between pure Mo and Mo14Re. On the one hand, high Re content further promotes the formation of Re–vacancy complexes, which tend to lower *S* and raise *W* parameter. On the other hand, the enhanced lattice distortion induced by high Re solid solution accelerates vacancy cluster growth, which tends to increase *S* and decrease *W*. The combination of these two opposing effects leads to a similar *S*–*W* distribution to that of pure Mo. Furthermore, the underlying defect characteristics evolve further into a Re–V–He complex defect system.

### 4.3. Chemical Environment at Defect Sites

**Figure 7:**
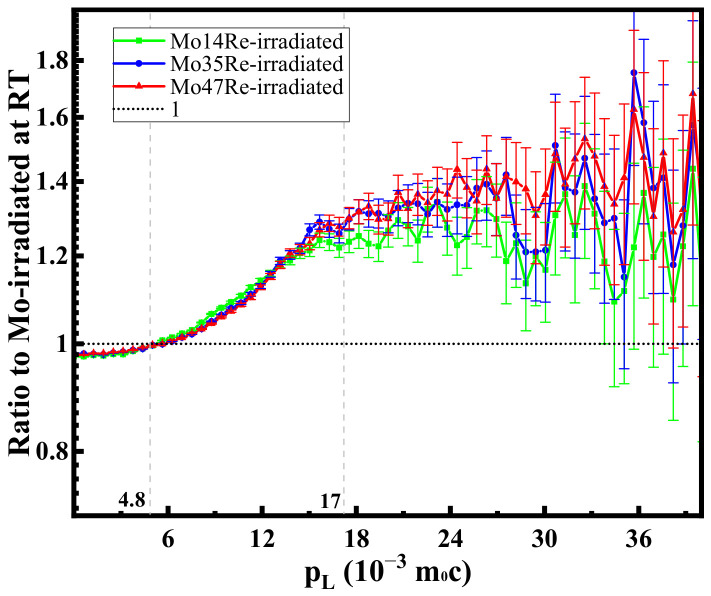
Conventional CDB results of Mo, Mo14Re, Mo35Re and Mo47Re alloys.

A conventional CDB technique was employed to characterize the evolution of local chemical environments surrounding bulk defects in irradiated Mo–Re alloys. Figure 7 presents the normalized CDB ratio curves of irradiated Mo14Re, Mo35Re, and Mo47Re relative to irradiated pure Mo. The results reveal that irradiated pure Mo possesses a relatively higher density of bulk vacancy-type defects compared with irradiated Mo–Re alloys. Accordingly, the corresponding ratio curves are slightly lower than unity in the low-momentum region (<6 × 10^−3^ m_0_c). No distinct characteristic peaks are observed in the high-momentum region. Given that conventional CDB measurements average signals over the entire sample depth, while irradiation damage is confined within a shallow layer below 1 μm, the majority of the sample matrix retains a low intrinsic defect level. Consequently, the interaction between Re solute atoms and vacancy-type defects cannot be effectively reflected by conventional CDB characterization.

**Figure 8:**
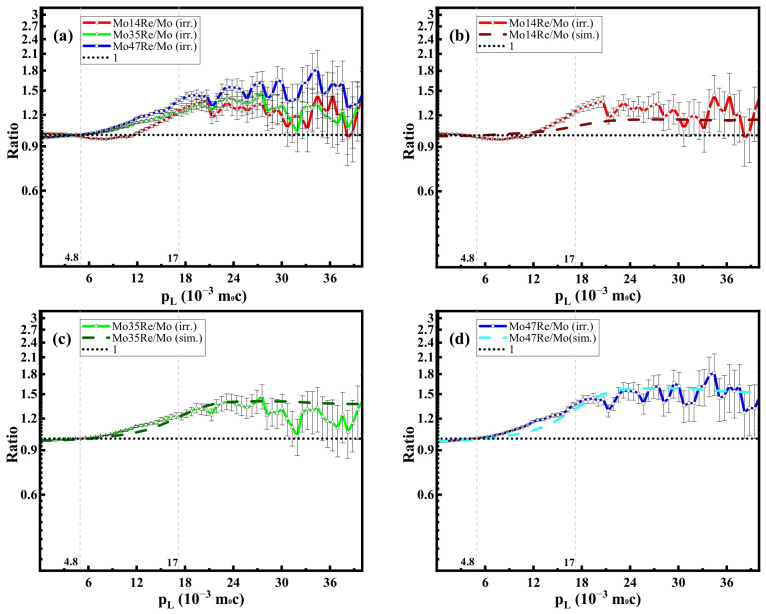
CDB ratio curves of irradiated Mo14Re, Mo35Re, and Mo47Re alloys normalized to irradiated Mo. Dashed lines are simulated CDB results. The x-axis is electron momentum component p_L_ (10^−3^ m_0_c). (**a**) Measured CDB ratios of three alloys; (**b**) Measured vs. simulated Mo14Re/Mo ratios; (**c**) Measured vs. simulated Mo35Re/Mo ratios; (**d**) Measured vs. simulated Mo47Re/Mo ratios. The dotted line at Ratio = 1 is the pure Mo reference.

To address this limitation, a coincidence Doppler broadening technique based on slow positron beam (SPB-CDB) was adopted to precisely characterize the local chemical environment surrounding defects at the specific irradiation depth of the specimens. Combined with the results in Figure 4b, the depth with the most significant differences in the *W* parameter among various specimens was selected, and a positron incident energy of 25 keV was determined accordingly. Under these test conditions, the normalized CDB ratio curves of irradiated Mo14Re, Mo35Re, and Mo47Re alloys relative to irradiated pure Mo were acquired, as presented in Figure 8.

According to reference [40], in all the one-dimensional momentum distribution results of SPB-CDB in Mo–Re alloys, two main regions can be distinguished: a low-momentum region (0 < p_L_ < 4.8 × 10^−3^ m_0_c, where p_L_ represents the longitudinal momentum of electrons), which reflects the annihilation of positrons with valence electrons; and a high-momentum region (4.8 × 10^−3^ m_0_c < p_L_ < 17 × 10^−3^ m_0_c), which arises from the annihilation of positrons with core electrons. These two regions convey information of the chemical environment and concentration of open-volume defects in Mo–Re alloys. The core electrons of each atom retain their atomic characteristics in solids. Thus, different atoms have different annihilation characteristic curves of their core electrons and positrons. The wave function of positrons trapped by defects overlaps with the atoms surrounding the defects, and these atoms can be identified by analyzing the high-momentum part of the annihilation spectrum. Therefore, it becomes possible to use the positron-core electron annihilation signal for chemical discrimination of atoms.

As shown in Figure 8a, at the depth of 675 nm, the concentration of irradiation-induced vacancy-type defects in various Mo–Re alloys is comparable to that in pure Mo with minor differences, and the CDB ratios remain around 1. In the high-momentum range, the ratios diverge gradually and increase with electron momentum, and finally stabilize within experimental uncertainty. First-principles calculations were performed to simulate the CDB curves of Mo–Re alloys normalized to pure Mo, while excluding the contribution of irradiation-induced defects. As shown in Figure 8b–d, Mo35Re and Mo47Re exhibit excellent consistency between experimental and simulated results. This indicates that the high-momentum electron density at defect sites in these alloys is consistent with the theoretical predictions under RT irradiation. Although the Mo14Re alloy presents a similar variation trend between experimental and simulated data, the experimental values are slightly higher than the simulated counterparts within the momentum range of 15–20 ×10^−3^ m_0_c, which is attributable to experimental measurement errors.

**Figure 9:**
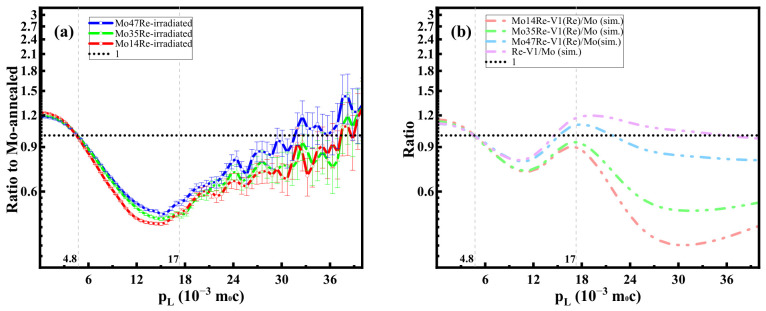
CDB ratio curves of irradiated Mo14Re, Mo35Re, and Mo47Re normalized to well-annealed pure Mo. (**a**) Experimental results; (**b**) Simulated CDB results of Mo–Re alloys with one single Re atom occupying a lattice vacancy, relative to pure Mo.

Figure 9 presents the normalized CDB curves of irradiated Mo–Re alloys relative to well-annealed pure Mo. This normalization method enables clearer characterization of the interaction between Re solutes and vacancy-type defects. As illustrated in Figure 9a, irradiated Mo–Re alloys exhibit a pronounced increase in electron density within the low-momentum region, with CDB ratios higher than unity. The elevation of ratio values in the low-momentum range inevitably leads to a reduction in the high-momentum region. Nevertheless, the relevant chemical information regarding Re atoms cannot be effectively extracted from the high-momentum signals.

Figure 9b shows the simulated CDB ratios of Mo–Re alloys where a single Re atom is replaced by a lattice vacancy. This microscopic model can reasonably represent the numerous vacancies substituting for Re atoms in the material. The simulation reveals that vacancy introduction remarkably raises the low-momentum ratio value above unity and correspondingly suppresses the high-momentum curves. Owing to the attractive interaction between Re atoms and vacancies, their binding energy is approximately 0.22–0.23 eV [41,42]. Therefore, Re atoms tend to interact with vacancies and form Re–vacancy complexes. The positron annihilation process near the complexes carries high-momentum electronic information derived from Re core electrons, producing a distinct Re characteristic peak around 17 × 10^−3^ m_0_c.

Compared with the simulation results, no obvious Re related peaks were observed in Figure 9a. Similar to previous PALS and DBS results, the SPB-CDB experiment confirmed that the macroscopic effect of Re on radiation-induced vacancy defects is limited under RT irradiation. Physically, due to mutual attraction, Re solute atoms can essentially interact with vacancies during irradiation, forming Re–vacancy complexes during the irradiation process. However, the low activation energy at room temperature is not sufficient to overcome the long-range migration barrier of these complexes. These defect solute structures are randomly dispersed throughout the matrix without long-range migration, continuous aggregation, or Re-rich nucleation and segregation. Therefore, the core electron annihilation signal distribution derived from Re is too weak to form distinguishable characteristic peaks in the CDB spectrum. In contrast, high-temperature irradiation provides sufficient thermal activation, enabling these complexes to effectively migrate and accumulate aggregates. The thermal driven defect evolution promotes significant Re enrichment and segregation at the defect site, forming a locally enriched Re microstructure, which can be manifested as distinct Re characteristic peaks in the SPB-CDB results. This work will be published in the future. The temperature dependent behavior originates from the thermal activation migration kinetics of solute defect complexes, similar to the high-temperature irradiation mechanism of Mo Re refractory alloys previously reported [18].

### 4.4. Defect Morphologies in the Radiation Damage Layer

**Figure 10:**
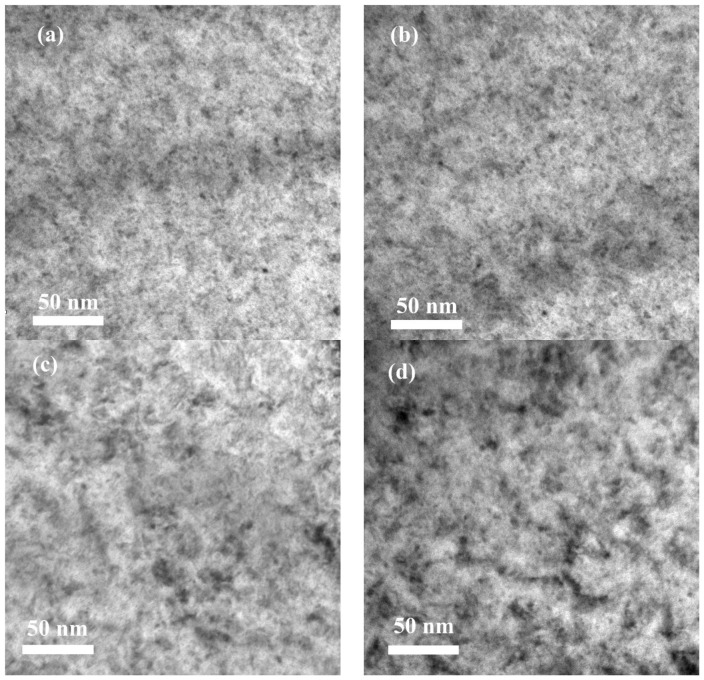
Cross-sectional TEM images showing the intrinsic defects in annealed (**a**) pure Mo, (**b**) Mo14Re, (**c**) Mo35Re and (**d**) Mo47Re alloys.

TEM characterization was carried out to analyze the microstructural defects and He distribution in pure Mo before and after irradiation. Figure 10 shows the bright-field TEM morphologies of annealed Mo, Mo14Re, Mo35Re and Mo47Re. The matrix displays uniform contrast without obvious dislocation entanglement or processing deformation, demonstrating that high-temperature annealing effectively relieves residual stress and stabilizes the microstructure.

**Figure 11:**
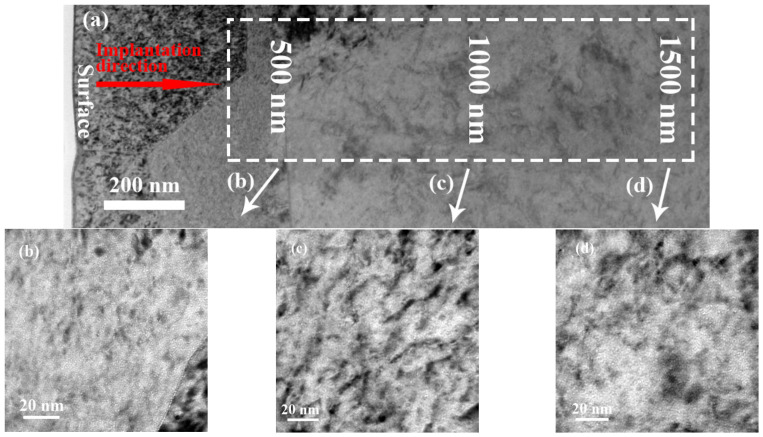
Cross-sectional TEM images showing the irradiated-induced at depths of 500–1500 nm along the implantation direction in Fe+Fe+He irradiated Mo. (**a**) Overview; (**b**) 500 nm; (**c**) 1000 nm; (**d**) 1500 nm.

**Figure 12:**
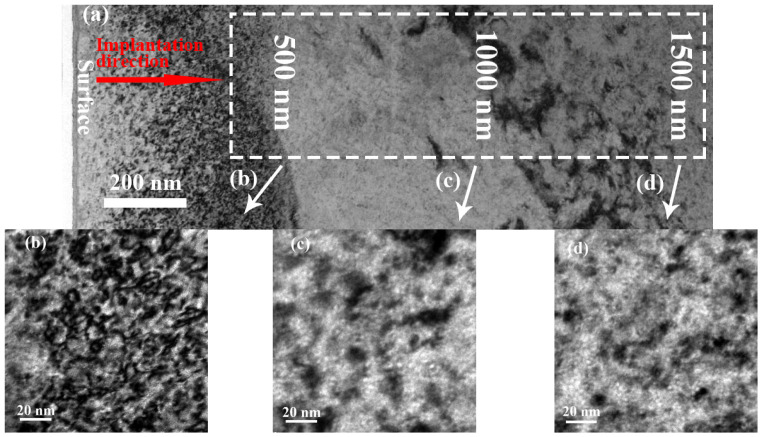
Cross-sectional TEM images showing the irradiated-induced at depths of 500–1500 nm along the implantation direction in Fe+Fe+He irradiated Mo14Re. (**a**) Overview; (**b**) 500 nm; (**c**) 1000 nm; (**d**) 1500 nm.

**Figure 13:**
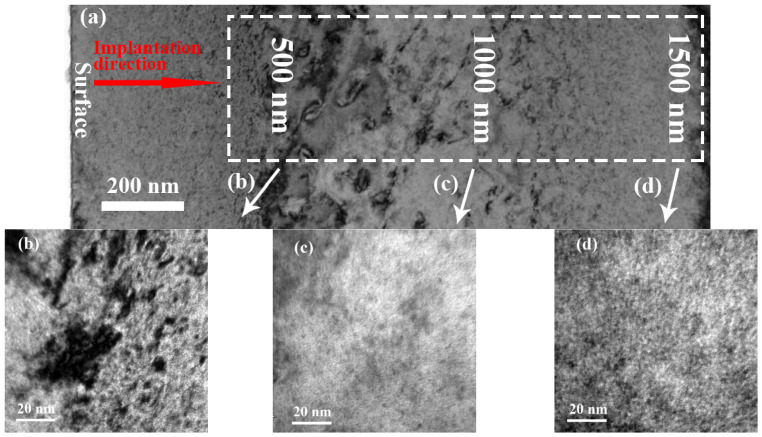
Cross-sectional TEM images showing the irradiated-induced at depths of 500–1500 nm along the implantation direction in Fe+Fe+He irradiated Mo35Re. (**a**) Overview; (**b**) 500 nm; (**c**) 1000 nm; (**d**) 1500 nm.

**Figure 14:**
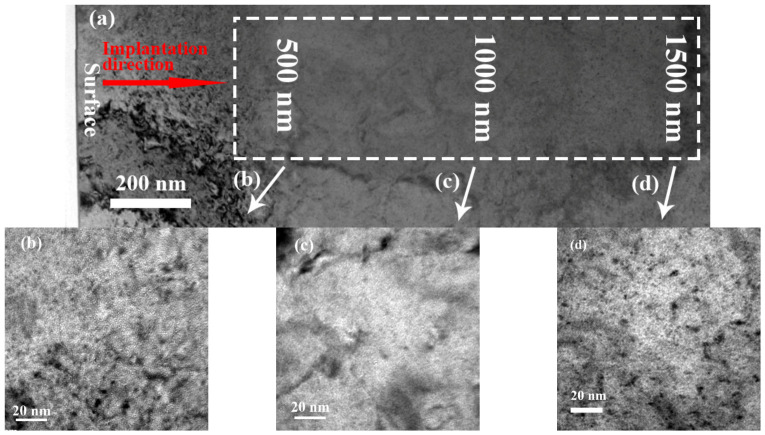
Cross-sectional TEM images showing the irradiated-induced at depths of 500–1500 nm along the implantation direction in Fe+Fe+He irradiated Mo47Re. (**a**) Overview; (**b**) 500 nm; (**c**) 1000 nm; (**d**) 1500 nm.

Figure 11, Figure 12, Figure 13 and Figure 14 present the microstructural characteristics of Mo, Mo14Re, Mo35Re, Mo47Re alloys after RT irradiation. Compared with annealed specimens, distinct cavity and dislocation structures are formed across the entire irradiated depth range, and these dark structures exhibit circular and elliptical morphologies. According to previous studies [43], cavities are usually the dominant damage structures after neutron irradiation, and dislocation loops can be observed at lower dpa levels. However, their number density is 1–2 orders of magnitude lower than that of cavities, and the distribution of cavities is generally governed by the sink effect of grain boundaries (GBs). Hu et al. [44] also reported that in a high-flux neutron reactor, large three-dimensional vacancy clusters are directly generated in pure tungsten by displacement cascades under neutron irradiation. Among these defects, nanoscale vacancy clusters with a diameter of 2.4 nm containing 150 vacancies were identified, and dislocation loops larger than 3.4 nm in diameter were also observed. High-magnification observations indicate that a large number of nearly spherical bright-white nanoparticles are produced after irradiation. According to the Fresnel contrast mechanism, gaseous bubble structures appear as bright spots under in-focus imaging conditions. During irradiation, He atoms preferentially aggregate and nucleate at matrix point defects for stable growth. He bubbles are densely distributed throughout the depth range of 500–1500 nm, with a size of 1–3 nm. It is noted that no obvious He bubble enrichment is observed in dark cavity regions of the matrix. In addition, He bubbles are dispersed not only inside the grains but also accumulated at grain boundaries, as shown in Figure 11b. Due to the lower diffusion energy barrier at grain boundaries, rapid diffusion channels are provided for the migration of He atoms. Irradiation-induced vacancies and interstitial He atoms tend to migrate and accumulate toward grain boundaries. The formation of stable He–vacancy complexes promotes the preferential nucleation and continuous growth of He bubbles at grain boundaries.

As shown in Figure 12, compared with pure Mo, the nanoscale He bubbles in the matrix of irradiated Mo14Re alloy exhibit significantly reduced density but increased size, with bubble sizes reaching up to 5 nm especially at a depth of approximately 1 μm. When the Re content is increased to 35 wt.% and 47 wt.% (Figure 13 and Figure 14), the behavior of He bubbles in Mo–Re alloys changes notably: within the near-surface region of 500 nm depth, the density of He bubbles increases significantly while the size decreases to 1–3 nm; at a depth of 1500 nm, the bubble size is further reduced to approximately 1 nm, showing a gradient distribution with depth.

The formation and evolution of He bubbles serve as direct indicators of potential irradiation-induced swelling in materials, and their size and density distributions are strongly dependent on the matrix composition. The present results show that pure Mo forms a high density of small and uniformly distributed He bubbles within a depth of 1.5 μm under RT irradiation. With the addition of 14 wt.% Re, Re atoms form stable solute–vacancy complexes with matrix vacancies, promoting the aggregation and coalescence of vacancies into larger vacancy clusters, which provide favorable conditions for the growth of He bubbles. Consequently, the size of He bubbles increases while their density decreases in the observed region, especially at depths greater than 1000 nm. When the Re content is further increased to 47 wt.%, the high concentration of Re atoms provides a larger number of stable vacancy trapping sites, inhibiting the long-range migration and coalescence of vacancies. This leads to preferential nucleation of He atoms at high-density small-size sites, resulting in a significant increase in He bubble density and a reduction in bubble size within the near-surface region of 500 nm. At depths greater than 1000 nm, the density of He bubbles decreases with the reduction in irradiation damage and He concentration, accompanied by a further reduction in bubble size.

In addition to He bubbles characterized by TEM, solute Re atoms potentially modulate other crystallographic defects such as dislocation loops, grain boundaries, and thermally activated σ/χ intermetallic precipitates in Mo–Re alloys, which cannot be comprehensively captured by positron annihilation measurements under the present RT irradiation condition. Previous neutron irradiation experiments have demonstrated that increasing Re content effectively suppresses the number density of irradiation-induced dislocation loops at moderate and elevated temperatures, and high Re concentrations promote preferential nucleation of brittle σ/χ precipitates along grain boundaries upon thermal activation [18]. Nevertheless, the RT irradiation adopted in this study fails to supply sufficient thermal driving force for long-range solute diffusion and stable precipitate nucleation, so precipitation-associated defects are scarcely generated within the measured irradiation depth, in good accordance with our TEM results showing no observable intermetallic precipitates. Furthermore, Re solutes tend to segregate at grain boundaries during prolonged irradiation via trapping vacancies and self-interstitials, altering grain-boundary cohesion and consequently the dislocation propagation pathway during plastic deformation [19]. Restricted vacancy long-distance mobility at ambient temperature hinders such grain-boundary segregation, rendering the influence of Re on grain-boundary configuration and dislocation evolution negligible in the present specimens. For high-temperature service environments of space reactor components, accelerated thermal diffusion facilitates diverse defect evolutions, and Re concentration turns into a dominant factor governing the development of dislocation networks and brittle precipitates apart from vacancy-type defects [45].

## 5. Conclusions

In this work, sequential heavy-ion and He-ion irradiations were carried out at RT to produce uniform damage defects in pure Mo, Mo14Re, Mo35Re, and Mo47Re alloys. Positron annihilation spectroscopy was used to characterize the size, concentration, depth distribution and local chemical environment of vacancy-type defects. The results indicated that

1. After RT irradiation, the longer lifetime component of Mo and Mo–Re alloys ranged from 262 to 280 ps, corresponding to medium-sized (2–6 vacancies) clusters.
2. A significant increase in the *S* parameter was observed owing to high-density vacancy-type defects, rising from 0.42 to approximately 0.50, while the differences among Mo–Re alloys remained relatively small (<0.01).
3. No high-momentum electronic signal originating from Re atoms was detected from the SPB-CDB results, whereas a distinct Re characteristic peak near 17 × 10^−3^ m_0_c was observed in the simulation.
4. The experimental results showed that the size, concentration, and depth distribution of vacancy-type defects in Mo–Re alloys were comparable to those of pure Mo. The inhibitory effect of Re atoms on the growth and depth accumulation of defects can be neglected under RT irradiation.

## Figures and Tables

**Table 1:** Elemental compositions of all specimens (wt.%).

Sample	Re	Matrix Mo	Impurities *	Total Impurities
Mo	≤0.01	99.99	≤0.01	≤0.01
Mo14Re	14 ± 2	Balance		
Mo35Re	35 ± 2.5	Balance		
Mo47Re	47 ± 3	Balance		

Notes *: Impurities include C, N, O, P, Bi, Sn, Al, Cu, Fe, Ni, Si, each ≤0.01 wt.%.

**Table 2:** Irradiation details.

Step	Ion	Energy	Flux	Fluence	Temperature
		MeV	Ions·cm^−2^·s^−1^	Ions·cm^−2^	K
I	Fe^13+^	3.5	2.4 × 10^11^	4.3 × 10^15^	RT
II	Fe^9+^	0.45	2.3 × 10^11^	1.3 × 10^15^	
III	He^2+^	0.16	4 × 10^12^	1 × 10^17^	

**Table 3:** PALS results of Mo, Mo14Re, Mo35Re and Mo47Re after RT irradiation, including the short-lifetime component $\tau_{1}$, long-lifetime component $\tau_{2}$, the third-lifetime component $\tau_{3}$, and the corresponding intensities $I_{1}$, $I_{2}$, $I_{3}$.

Sample	$\tau_{1}$ (ps)	$I_{1}$ (%)	$\tau_{2}$ (ps)	$I_{2}$ (%)	$\tau_{3}$ (ps)	$I_{3}$ (%)
Mo	131.8 ± 1.1	80.45 ± 0.98	261.6 ± 5.2	18.93 ± 0.99	2310.0 ± 96	0.62 ± 0.02
Mo14Re	121.0 ± 1.0	85.51 ± 0.67	277.8 ± 6.8	13.50 ± 0.68	1944.0 ± 47	0.99 ± 0.03
Mo35Re	123.4 ± 0.5	87.41 ± 0.49	280.1 ± 5.9	11.91 ± 0.49	2317.0 ± 99	0.67 ± 0.02
Mo47Re	126.3 ± 1.6	85.00 ± 1.40	266.6 ± 9.6	14.40 ± 1.40	2180.0 ± 100	0.61 ± 0.03

**Figure A1:**
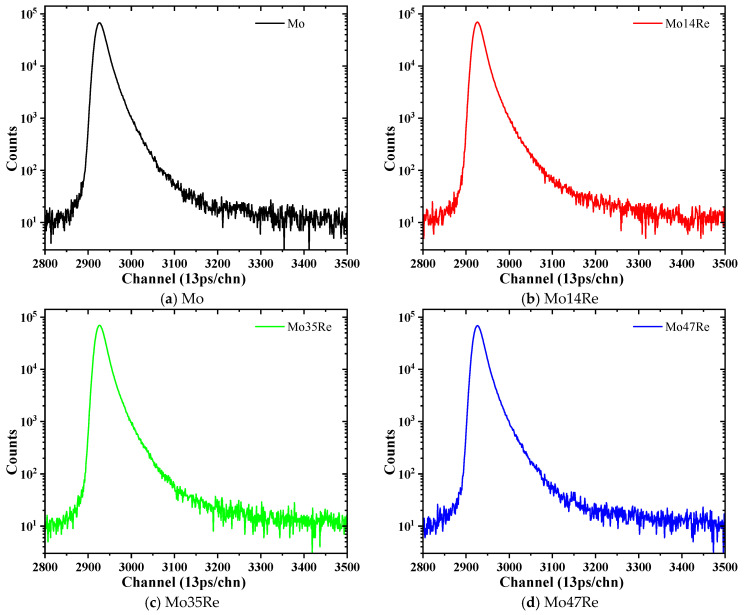
Raw PAL spectra of (**a**) pure Mo, (**b**) Mo14Re, (**c**) Mo35Re, and (**d**) Mo47Re alloys after RT ion irradiation.

## Data Availability

The original contributions presented in this study are included in the article. Further inquiries can be directed to the corresponding authors.
